# Diagnostic and management challenges of prune belly syndrome in a low-income country: a neonatal case report

**DOI:** 10.1093/jscr/rjaf824

**Published:** 2025-10-21

**Authors:** Mohamed Nur Ali, Abdisalam Ismail Hassan, Ali Abdi Jama, Abdisalam Mohamed Sh Abdilahi, Abdisamad Omar Ali, Fardowsa Hassan Ahmed, Yasir Khalif Ali, Farah Ali Ahmed, Farah Abdullahi Ismail, Fardowso Ali Mohamud, Ahmed Mohamed Ali, Ismail Gedi Ibrahim

**Affiliations:** Pediatric Department, Mogadishu Somali-Türkiye Recep Tayyip Erdoğan Training and Research Hospital, Digfer Street, Hodan District, Mogadishu, Somalia; Faculty of Medicine and Health Sciences, Jamhuriya University of Science and Technology, Digfer Street, Hodan District, Mogadishu, Somalia; Faculty of Medicine and Health Sciences, Jamhuriya University of Science and Technology, Digfer Street, Hodan District, Mogadishu, Somalia; Pediatric Department, Mogadishu Somali-Türkiye Recep Tayyip Erdoğan Training and Research Hospital, Digfer Street, Hodan District, Mogadishu, Somalia; Pediatric Department, Mogadishu Somali-Türkiye Recep Tayyip Erdoğan Training and Research Hospital, Digfer Street, Hodan District, Mogadishu, Somalia; Pediatric Surgery Department, Mogadishu Somali-Türkiye Recep Tayyip Erdoğan Training and Research Hospital, Digfer Street, Hodan District, Mogadishu, Somalia; Pediatric Department, Mogadishu Somali-Türkiye Recep Tayyip Erdoğan Training and Research Hospital, Digfer Street, Hodan District, Mogadishu, Somalia; Pediatric Department, Mogadishu Somali-Türkiye Recep Tayyip Erdoğan Training and Research Hospital, Digfer Street, Hodan District, Mogadishu, Somalia; Pediatric Department, Mogadishu Somali-Türkiye Recep Tayyip Erdoğan Training and Research Hospital, Digfer Street, Hodan District, Mogadishu, Somalia; Pediatric Department, Mogadishu Somali-Türkiye Recep Tayyip Erdoğan Training and Research Hospital, Digfer Street, Hodan District, Mogadishu, Somalia; Pediatric Department, Mogadishu Somali-Türkiye Recep Tayyip Erdoğan Training and Research Hospital, Digfer Street, Hodan District, Mogadishu, Somalia; Infectious Disease and Clinical Microbiology Department, Mogadishu Somali-Türkiye Recep Tayyip Erdoğan Training and Research Hospital, Digfer Street, Hodan District, Mogadishu, Somalia; Radiology Department, Mogadishu Somali-Türkiye Recep Tayyip Erdoğan Training and Research Hospital, Digfer Street, Hodan District, Mogadishu, Somalia

**Keywords:** prune belly syndrome, cryptorchidism, hydronephrosis, nephrostomy, abdominal distention, a case report

## Abstract

Prune Belly Syndrome (PBS) is a rare congenital anomaly defined by deficient abdominal wall musculature, urinary tract abnormalities, and cryptorchidism. It is associated with high morbidity, particularly in low-resource settings where prenatal imaging and specialized surgical care are limited. We report the first documented case of PBS from Somalia in a 6-day-old male neonate delivered without antenatal care. He presented with abdominal distension, respiratory distress, bilateral undescended testes, and oliguria. Laboratory tests showed impaired renal function and elevated inflammatory markers. Ultrasound revealed multilocular cystic hydronephrosis with thinned renal parenchyma, and a nephrostogram confirmed bilateral ureteropelvic junction obstruction. The infant was managed with oxygen, antibiotics, and bilateral percutaneous nephrostomy, which led to improved urine output, normalized renal function, and resolution of abdominal distension. He was discharged in stable condition after 15 days. This case underscores the variability of urinary tract pathology in PBS and highlights the importance of early recognition and timely surgical intervention to preserve renal function in resource-limited regions.

## Introduction

Prune Belly Syndrome (PBS), also called Eagle–Barrett or Obrinsky syndrome, is a rare congenital disorder defined by a triad of deficient abdominal wall musculature, urinary tract anomalies, and cryptorchidism [1]. Its etiology remains unclear, though genetic factors have been suggested, including associations with trisomy 21 and chromosome 6q deletions [2]. PBS occurs in ~3.6–3.8 per 100 000 live births, with males comprising 95% of cases [3].

Affected neonates display a characteristic ‘prune-like’ abdomen and may have multisystem involvement—cardiopulmonary (≈49%), musculoskeletal (≈65%), and gastrointestinal (≈63%) anomalies [4]. Prognosis depends on renal function and pulmonary development; perinatal mortality ranges from 10%–25% [4]. Females are rarely affected and generally lack gonadal anomalies [5].

In sub-Saharan Africa, PBS is exceedingly rare, with many pediatricians never encountering a case during their careers [6, 7]. We present a rare documented Somali case of PBS, underscoring the diagnostic and management challenges in a low-resource setting.

## Case presentation

A 6-day-old Somali male neonate was delivered through normal vaginal delivery in Cadaado, Somalia, and referred to our hospital for further assessment of insufficient abdominal wall musculature, abdominal distention, low urine output, and respiratory distress. He was lethargic and tachycardic on arrival due to hypoglycemia (blood glucose 37 mg/dl, corrected with 2 mg/kg of 10% dextrose). The mother was 27 years old (gravida 3, para 3) with no medical history, and the pregnancy was not screened by ultrasound or laboratory tests. Family history was negative for congenital disorders.

**Figure 1:**
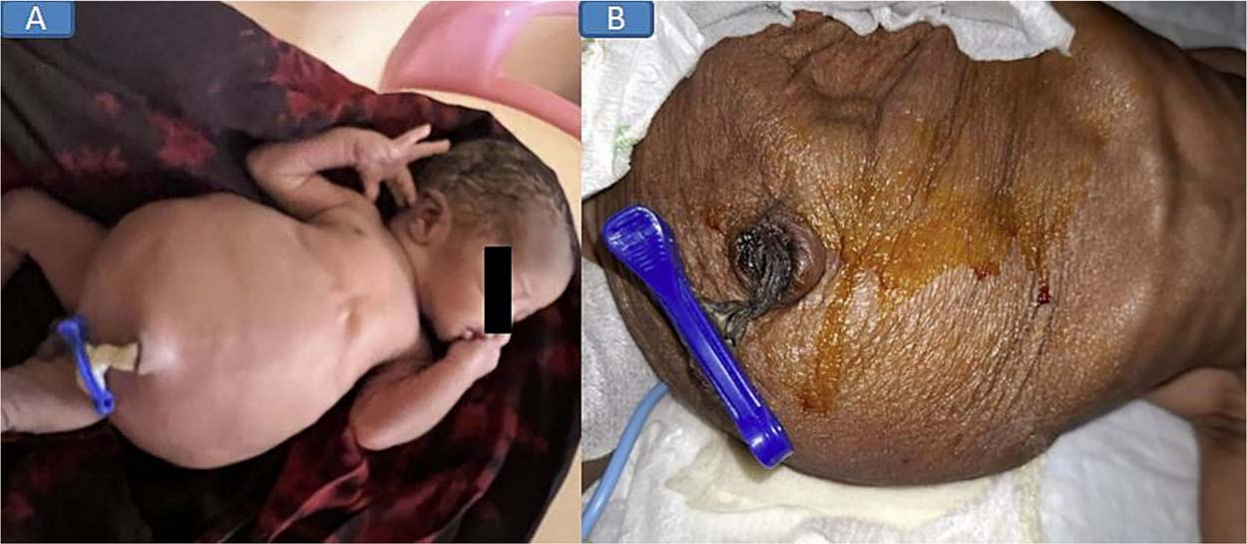
Pre-nephrostomy drainage abdominal distention (A) and post-nephrostomy (B).

On examination, the neonate weighed 2.905 kg, measured 45 cm in length, and had a head circumference of 32 cm, all appropriate for gestational age. He was lethargic, in respiratory distress requiring continuous positive airway pressure (CPAP) oxygen support, and had a soft, distended, prune-like abdomen (Fig. 1). The anal canal was patent, and stool passage was normal. Both scrotal sacs were empty.

Laboratory results revealed elevated creatinine (1.46 mg/dl), urea (76 mmol/L), and markedly high C-reactive protein (CRP) (142 mg/dl). Human immunodeficiency virus (HIV), hepatitis B virus (HBV), and hepatitis C virus (HCV) were negative.

**Figure 2:**
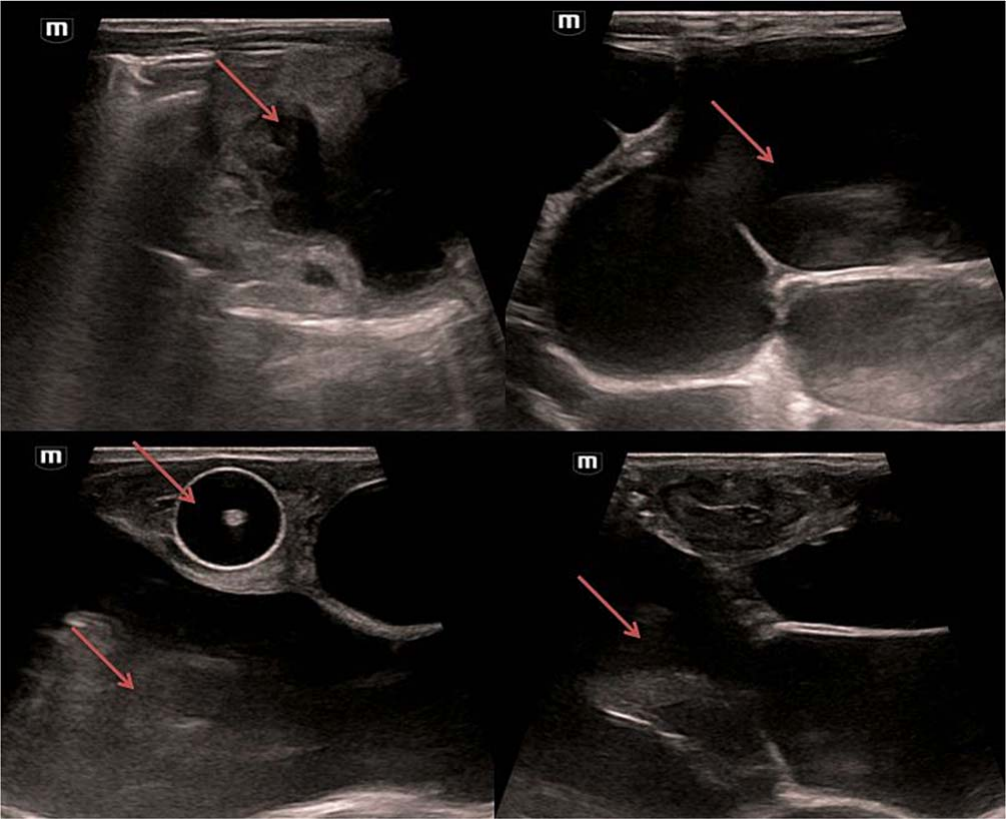
Bilateral multilocular cystic hydronephrosis.

**Figure 3:**
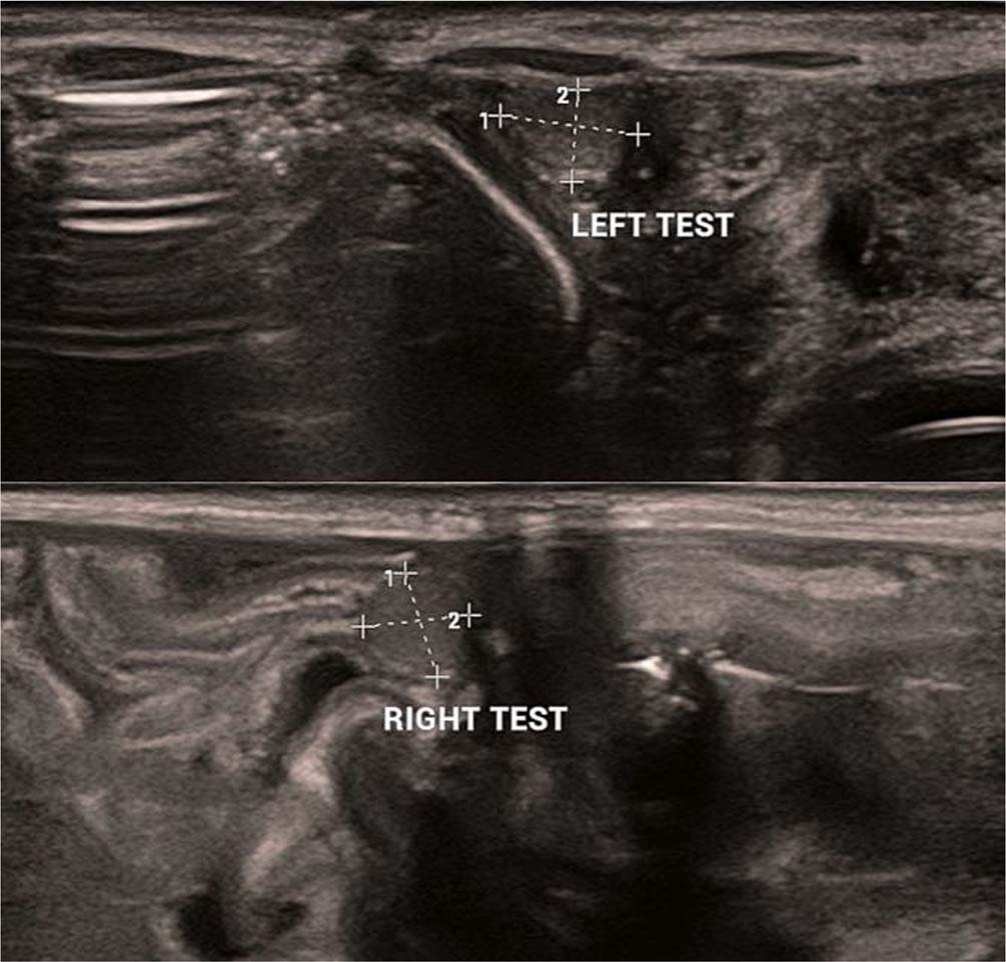
Bilateral cryptorchidism.

**Figure 4:**
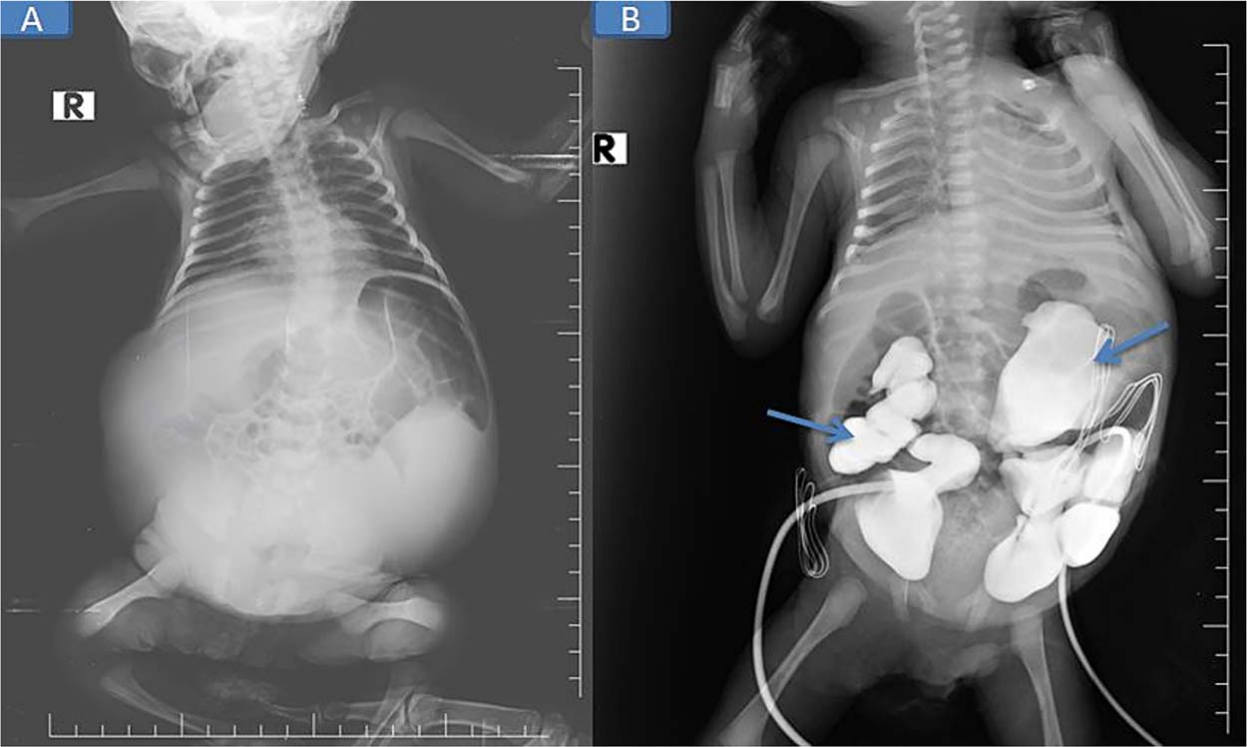
Pre-op abdominal X-ray (A) and post-operative percutaneous nephrostomy procedure with contrast injection through the nephrostomy (B).

Abdominal and scrotal ultrasonography demonstrated multilocular cystic hydronephrosis with thinning of renal parenchyma, extending into the pelvis, and absence of testes within the scrotum. Both testes were located intra-abdominally between bowel loops (left 7 × 4.5 mm, right 6 × 5.5 mm) (Figs 2 and 3). Abdominal X-ray after percutaneous nephrostomy with contrast revealed pooling in both kidneys without ureteric passage, suggesting bilateral ureteropelvic junction obstruction (UPJO) (Fig. 4).

The patient was stabilized with CPAP oxygen, intravenous antibiotics (ampicillin, cefotaxime, metronidazole), and supportive care. Following preoperative evaluation and parental consent, bilateral nephrostomy was performed, yielding significant urine drainage. Postoperatively, renal function improved with normalization of creatinine and urea, and by Day 5 post-nephrostomy, values had returned to normal (urea 13 mg/dl [reference: 10–45] and creatinine 0.48 mg/dl [reference: 0.35–1.10]), accompanied by a reduction in abdominal distention.

He tolerated breastfeeding well and was discharged after 15 days in good condition, with ongoing outpatient follow-up at the pediatric surgery clinic.

## Discussion

PBS affects 3.6–3.8 per 100 000 live births, predominantly males [3, 8]. Its hallmark, a prune-like abdomen, results from absent or hypoplastic abdominal musculature. Cryptorchidism and urinary tract anomalies, including hydronephrosis and UPJO, form the diagnostic triad.

Pathogenesis remains debated. One hypothesis links PBS to mesodermal developmental defects between 6 and 10 weeks’ gestation [10]. Severe obstructive uropathy and urinary ascites may contribute to abdominal wall muscle degeneration and failed testicular descent [9].

Diagnosis is usually antenatal via ultrasound, typically in the second trimester. In this case, absence of antenatal imaging in rural Somalia delayed recognition until postnatal presentation with abdominal distension and renal dysfunction. Postnatal ultrasound and contrast nephrostogram confirmed bilateral UPJO, a key distinguishing feature.

Prognosis depends largely on renal and pulmonary function. Favorable outcomes are seen when at least one kidney functions normally and serum creatinine remains <0.7 mg/dl during childhood [4]. Our patient improved significantly following nephrostomy, normalizing renal function before discharge.

Management requires a multidisciplinary approach: pediatric urologists, nephrologists, pulmonologists, gastroenterologists, and physical therapists coordinate long-term care [11]. In resource-limited settings, this ideal is often constrained by infrastructure and finances. Our case demonstrates that timely decompression of obstructed kidneys via nephrostomy can stabilize critically ill neonates, even when definitive reconstructive urologic surgery is delayed.

Comparison with literature: Previously reported Somali cases described severe jaundice and vesicoureteral reflux managed conservatively due to financial limitations. In contrast, our case was distinguished by confirmed bilateral UPJO treated successfully with nephrostomy, underscoring the importance of individualized management even in constrained environments.

## Conclusion

PBS is a rare, life-threatening congenital disorder with high morbidity and mortality. Early antenatal detection and multidisciplinary management optimize outcomes. In resource-limited settings such as Somalia, delayed diagnosis and constrained surgical options pose significant challenges. This case demonstrates that timely supportive care, including nephrostomy, can improve renal function and short-term survival. More research is needed to better understand PBS genetics, optimize management, and improve access to advanced pediatric urology in low-income countries.

## Data Availability

Available from the corresponding author upon request.
